# NKAP alters tumor immune microenvironment and promotes glioma growth via Notch1 signaling

**DOI:** 10.1186/s13046-019-1281-1

**Published:** 2019-07-06

**Authors:** Guangyan Gu, Taihong Gao, Lu Zhang, Xiuyang Chen, Qi Pang, Yanan Wang, Dan Wang, Jie Li, Qian Liu

**Affiliations:** 1 Department of Histology and Embryology, School of Basic Medical Science, Shandong University Cheeloo College of Medicine, 44# Wenhua Xi Road, Jinan, 250012 Shandong People’s Republic of China; 2 Department of Neurosurgery, Shandong Provincial Hospital Affiliated to Shandong University, Jinan, 250021 Shandong China; 3 Department of Peripheral Vascular Disease, Affiliated Hospital of Shandong University of Traditional Chinese Medicine, Jinan, Shandong China; 4 Assisted Reproductive Centre, Shandong Maternity and Child Health Care Hospital, Jinan, Shandong China

**Keywords:** NKAP, Notch1, TAM, Glioma

## Abstract

**Background:**

Glioma is one of the most aggressive malignant brain tumors which is characterized with highly infiltrative growth and poor prognosis. NKAP (NF-κB activating protein) is a widely expressed 415-amino acid nuclear protein that is overexpressed by gliomas, but its function in glioma was still unknown.

**Methods:**

CCK8 and EDU assay was used to examine the cell viability in vitro, and the xenograft models in nude mice were established to explore the roles of NAKP in vivo. The expressions of NKAP, Notch1 and SDF-1 were analyzed by immunofluorescence analysis. The expression of NKAP and Notch1 in glioma and normal human brain samples were analyzed by immunohistochemical analysis. In addition, CHIP, Gene chip, western blot, flow cytometry, immunofluorescence, ELISA and luciferase assay were used to investigate the internal connection between NKAP and Notch1.

**Results:**

Here we showed that overexpression of NKAP in gliomas could promote tumor growth by contributing to a Notch1-dependent immune-suppressive tumor microenvironment. Downregulation of NKAP in gliomas had abrogated tumor growth and invasion in vitro and in vivo. Interestingly, compared to the control group, inhibiting NKAP set up obstacles to tumor-associated macrophage (TAM) polarization and recruitment by decreasing the secretion of SDF-1 and M-CSF. To identify the potential mechanisms involved, we performed RNA sequencing analysis and found that Notch1 appeared to positively correlate with the expression of NKAP. Furthermore, we proved that NKAP performed its function via directly binding to Notch1 promoter and trans-activating it. Notch1 inhibition could alleviate NKAP’s gliomagenesis effects.

**Conclusion:**

these observations suggest that NKAP promotes glioma growth by TAM chemoattraction through upregulation of Notch1 and this finding introduces the potential utility of NKAP inhibitors for glioma therapy.

**Electronic supplementary material:**

The online version of this article (10.1186/s13046-019-1281-1) contains supplementary material, which is available to authorized users.

## Background

Glial-derived gliomas account for the vast majority of malignant brain tumors [1]. Researches have shown that 27% of all brain tumors and 80% of all malignant brain tumors in the USA are gliomas [2, 3]. Malignant gliomas have an incidence rate of 5.26 per 100,000 inhabitants, and approximately 17,000 new cases are diagnosed per year. The National Cancer Institute (NCI) estimated that brain malignancies constitute 23,800 cases, with 16,700 deaths per year [4]. Gliomas are classified as grade I to IV depending on their histopathological and genetic characteristics [5]. Considering the unfavorable prognosis and poor quality of life associated with gliomas, clarifying the molecular mechanisms would provide a theoretical basis for developing effective treatment strategies or identifying new therapeutic targets.

NKAP (NF-κB activating protein) is a widely expressed 415-amino acid nuclear protein that is evolutionarily conserved in mammals [6]. Self-evidently, NKAP was first discovered in inflammatory and immunological process. Recently, some studies showed that NKAP played a more important role in immune system via inhibiting Notch-mediated transcription instead of NF-kB signaling. Loss of NKAP transcriptionally activated Notch target genes and blocked αβ T cell development at the double-negative 3 (DN3) to double-positive (DP) transition [8]. Besides NKAP was crucial for invariant natural killer T (iNKT) cell development [9] as well as their proliferation and differentiation into ROR-γt-expressing NKT17 cells [10]. Additional to the functions in the immune system, NKAP was critical for hematopoietic stem cell maintenance and [11] neurogenesis [12]. Knockdown of the *Drosophila melanogaster* gene CG6066, an NKAP ortholog, led to over proliferation of *D. melanogaster* neural precursor cells, resulting in lethal tumor formation [13]. On the other hand, in the mammals NKAP was almost ubiquitously expressed throughout the brain and is strongly expressed in progenitor cells near the subventricular zone (SVZ) neural stem cell (NSC) niche but lowly expressed in glial cells and differentiated cells. Its expression at different positions in the brains of mice was consistent with Notch1 expression during the process of neurogenesis [13]. Considering increased expression of stemness related genes are usually pronounced in the malignancy, the role of NKAP in tumors, especially in nervous system tumors, has never been reported.

As mentioned above, in addition to NF-κB signaling, the most widely reported molecule targeted by NKAP is the Notch receptor. It is well known that the Notch signaling pathway plays important roles in different tissue developmental processes, such as cell differentiation, survival, and proliferation [14], and is also involved in tumorigenesis [15], such as cervical, colon, head and neck, lung, and renal carcinoma and pancreatic and breast cancer [16]. Notch1 signaling is activated via juxtacrine binding of an adjacent cell’s Delta-like or Jagged ligand. Then, the Notch intracellular domain (NICD) translocates into the nucleus and binds to members of the CSL transcription factor family [17]. The function of Notch signaling in tumorigenesis, either oncogenic or tumor-suppressive, largely depends on the cellular context. Previous research has shown that Notch1 is upregulated in many glioma cell lines and primary human gliomas. It promotes glioma cell survival, proliferation, migration and invasion [18, 19]. Notch1 is also associated with tumor progression [20, 21]. In particular, increased expression of Notch1 is correlated with increasing grades of glioma malignancy [22].

In this study, we provided the first evidence showing the functional roles of NKAP in gliomas by targeting Notch1 signaling. Although NKAP inhibited the Notch1 downstream pathway in the immune system, our study revealed that it activated the Notch signaling in gliomas. NKAP silencing significantly inhibited the proliferation, migration and invasion of glioma cells, whereas overexpression of NKAP induced aggressive cellular behavior. We also observed that NKAP played the same role in vivo as in vitro. Additionally, the abovementioned effects of NKAP were achieved by targeting Notch1 signaling. When the Notch1 pathway was inhibited by RNA interference, the effects induced by upregulating NKAP were reduced. Most importantly, we found that NKAP could alter the polarization and infiltration of tumor associated macrophages (TAM) via regulating secretion of SDF-1 and M-CSF, indicating that NKAP might contribute to the immune microenvironment of gliomas. Taken together, it is concluded that NKAP performs its oncogenic functions via Notch1 signaling, and this finding provides a novel perspective to find potential therapeutic targets for gliomas.

## Experimental methods

### Cell lines and tissue samples

Glioma cell lines U251, U87 and Gl261 were obtained from the Cell Bank of Type Culture Collection of Chinese Academy of Sciences (Shanghai, China) and cultured in Dulbecco’s modified Eagle’s medium (DMEM; HyClone, Logan, UT, USA) with high glucose and sodium pyruvate, supplemented with 12% fetal bovine serum (FBS; Gibco, Life Technologies, Carlsbad, CA, USA), 100 units/mL penicillin and 100 μg/mL streptomycin (Invitrogen, Life Technologies) at 37 °C with 5% CO2. 90 GBM specimens and 12 normal human brain specimens were obtained from the Department of Neurosurgery at Provincial Hospital affiliated to Shandong University. Ethical approval was obtained from Shandong University Ethics Committee, and all of the patients provided written informed consents.

### Cells transfection with lentiviruses

Lentiviruses carrying shRNA targeting human NKAP lentiviral vectors (the pGCSIL-GFP-shRNA-NKAP or pLKD-CMV-Puro-U6-shRNA-NKAP) were from GeneChem. U87, U251 or GL261 cells (2 × 105 per well) were cultured in a six well plate the day before transfection. The lentiviruses or siRNA were transfected into the cells, according to the manufacturers’ introduction. siRNA target sequence is GACGAAAGAGAGAGAACAA.

### Immunohistochemistry (IHC)

Tissue samples were fixed in 4% paraformaldehyde, paraffin embedded and serially sectioned into 5-μm-thick sections. IHC staining was performed using the standard avidin-biotin complex method. The primary antibodies used in the study were as follows: anti-NKAP (Abcam, Cambridge, MA, USA) and anti-Notch1 (Notch intracellular domain 1, NICD1) (Cell Signaling and Technology, Boston, MA, USA). Immunohistochemistry results were evaluated by a semiquantitative approach used to assign an H-score (or “histo” score). Firstly, the staining score was determined by the intensity of positive staining (no staining = 0; weak staining = 1; moderate staining = 2; strong staining = 3). Then the percentage of cells at each staining intensity level was calculated. An H-score was assigned using the following formula: [1 × (% cells 1+) + 2 × (% cells 2+) + 3 × (% cells 3+)]. The H score, ranging from 0 to 300, represented higher weight for higher-intensity staining in a given sample. In this study, the median of H score is 157.

### Western blot analysis and ELISA

Total proteins were extracted using lysis buffer containing 10 mmol/L Tris-HCl (pH 7.4), 1% Triton X-100 and protease/phosphates inhibitors (Roche Diagnostics, Indianapolis, IN, USA), separated by 10% SDS-PAGE gel electrophoresis, transferred to polyvinylidene difluoride (PVDF) membranes and probed with primary antibodies. The membranes were subsequently probed with horseradish peroxidase-conjugated secondary antibodies followed by development using an enhanced chemiluminescence detection system (Pierce, Rockford, IL, USA). Anti-GAPDH antibody was used to monitor the loading amount. M-CSF ELISA were performed according to the manufacturer’s instructions (Abcam, USA).

### Quantitative reverse transcription PCR (qRT-PCR)

Trizol reagent (Gibco, Birmingham, MI, USA) was used to extract RNA. The concentration and purity of RNA were determined by measuring the absorbance at 260 nm and the absorbance ratio of 260/280 nm in a Nano-Drop 8000 Spectrophotometer (Thermo Scientific, Wilmington, DE, USA). A PrimeScript RT reagent kit with gDNA Eraser (Takara, Japan) was used to synthesize the cDNA. An ABI 7300 Fast Real-time PCR System (Applied Biosystems, Carlsbad, CA, USA) and an SYBR Green PCR kit (Applied Takara, Japan) were used for real-time PCR. The primer sequences were as follows:

- NKAP Forward 5′-GGATCCTCACTTGTCATCCTTCCCTTTG-3′.
  Reverse 5′-GAATTCATGGCTCCTGTATCGGGCTC -3′.
- NOTCH1 Forward 5′-AAGCTGCATCCAGAGGCAAAC-3′.
  Reverse 5′-TGGCATACACACTCCGAGAACAC-3′.
- NOTCH2 Forward 5′-GTTACAGCAGCCCTTGCCTGA-3′.
  Reverse 5′-CCATGGATACAAGGGTTACTTGCAC-3′.
- NOTCH3 Forward 5′-ATCGGCTCGGTAGTAATGCTG-3′.
  Reverse 5′-ACAACGCTCCCAGGTAGTCA-3′.
- NOTCH4 Forward 5′-TGCGAGGAAGATACGGAGTG-3′.
  Reverse 5′-GGACGGAGTAAGGCAAGGAG-3′.
- CCND1 Forward 5′-GGGCCACTTGCATGTTCGT-3′.
  Reverse 5′-CAGGTTCCACTTGAGCTTGTTCAC-3′.
- CTNNB1 Forward 5′-GAGTGCTGAAGGTGCTATCTGTCT-3′.
  Reverse 5′-GTTCTGAACAAGACGTTGACTTGGA-3′.
- DVL2 Forward 5′-GACATGAACTTTGAGAACATGAGC-3′.
  Reverse 5′-CACTTGGCCACAGTCAGCAC-3′.
- HES1 Forward 5′-GGACATTCTGGAAATGACAGTGA-3′.
  Reverse 5′-AGCACACTTGGGTCTGTGCTC-3′.
- N-cadherin Forward 5′-CTCCTATGAGTGGAACAGGAACG-3′.
  Reverse 5′-TTGGATCAATGTCATAATCAAGTGCTGTA-3′.
- Twist1 Forward 5′-AGCTACGCCTTCTGGTCT-3′.
  Reverse 5′-CCTTCTCTGGAAACAATGACATC-3′.
- Vimentin Forward 5′-AGATCGATGTGGACGTTTCC-3′.
  Reverse 5′-CACCTGTCTCCGGTATTCGT-3′.
- SDF-1 Forward 5′- TCTCCATCCACATGGGAGCCG-3′.
  Reverse 5′- GATGAGGGCTGGGTCTCACTCTG-3′.
- GAPDH Forward 5′-GCACCGTCAAGGCTGAGAAC-3′.
  Reverse 5′-TGGTGAAGACGCCAGTGGA-3′.

### RNA sequencing

Total RNA was extracted using Trizol (Invitrogen) and treated with DEPC water. After RNA quality examination, A total amount of 2 μg RNA per sample was used as input material for the RNA sample preparations. Sequencing libraries were generated using NEBNext® Ultra™ RNA Library Prep Kit for Illumina® (#E7530L, NEB, USA) following the manufacturer’s recommendations and index codes were added to attribute sequences to each sample. RNA concentration of library was measured using Qubit® RNA Assay Kit in Qubit® 3.0 to preliminary quantify and then dilute to 1 ng/μl. Insert size was assessed using the Agilent Bioanalyzer 2100 system (Agilent Technologies, CA, USA), and qualified insert size was accurate quantification using StepOnePlus™ Real-Time PCR System (Library valid concentration>10 nM). The clustering of the index-coded samples was performed on a cBot cluster generation system using HiSeq PE Cluster Kit v4-cBot-HS (Illumina) according to the manufacturer’s instructions. After cluster generation, the libraries were sequenced on an Illumina Hiseq 4000 platform and 150 bp paired-end reads were generated. The mRNA sequencing assay was achieved by Annoroad Gene Technology Co., Ltd., Beijing, China.

### Cell proliferation

Cell proliferation was determined using a Cell Counting Kit-8 (CCK-8) assay kit (Dojindo, Japan) and a cell-light 5-ethynyl-2′-deoxyuridine (EdU) Apollo Imaging Kit (Ribobio, Guangzhou, China). For the CCK-8 assay, U87 and U251 cells were seeded into 96-well plates for 0, 24, 48, and 72 h at a density of 3000 cells per well. Then, 10 μL CCK-8 solution was added to each well and incubated with the cells for 2 h. Absorbance was detected at 450 nm using a microplate reader (Bio-Rad, Hercules, CA, USA). EdU immunocytochemistry staining was performed by using a Cell-Light™ EdU Apollo In Vitro Imaging Kit (Ribobio, Guangzhou, China) at 24 h after the cell was plated into 96-well plates. The EdU-positive cells were visualized under a fluorescence microscope (Olympus, Tokyo, Japan).

### Cell migration and invasion assay

To assess the migration and invasion ability of glioma cells in vitro, migration and invasion assays were performed using transwell chambers with 8-μm pores (Corning star, Lowell, MA, USA). For the migration assay, 1000 transfected cells were suspended in 200 μL serum-free medium and added to the upper transwell chamber. After incubation for 12 h in a humidified atmosphere containing 5% CO_2_ at 37 °C, the migrated cells that had stuck to the lower surface of the membrane were fixed in 4% paraformaldehyde and stained with 0.1% crystal violet for 5 min. The number of migrated cells was counted in five randomly selected fields at 200× magnification using a microscope. For the invasion assay, the transwell chambers were coated with Matrigel (BD Bioscience), and same procedures as those for the migration assay were followed.

### Luciferase reporter assay

Cells were plated in 48-well plates, transfected with the reporter plasmid pGL2-Notch1 promoter-Luc and together with an siRNA-NKAP or control expression vector. Luc activities were determined using a luciferase assay system (Promega, Madison, WI, USA) over a period of 24 h.

### Chromatin immunoprecipitation (ChIP)

U87 cells were cross-linked with 1% formaldehyde and quenched by adding 125 mM glycine. Chromatin was isolated by adding cell lysis buffer (1% SDS, 10 mM EDTA, 50 mM Tris-HCl, pH 8.1, 1 mM PMSF), and DNA were sheared into fragments of 300–500 bp by sonication. Lysates were pre-cleared for 1–2 h using Salmon Sperm DNA/Protein A Agarose (EMD Millipore, Billerica, MA, USA), after which precipitation was induced using anti-H3K27me3 (Cell Signaling and Technologies, Boston, MA, USA) or anti-NKAP (Abcam). An isotype-matched IgG was used as a negative control. To reverse the DNA cross-linking, the precipitates were incubated with pronase for 2 h at 42 °C and 68 °C for 8 h. The Notch1 promoter DNA in the immunoprecipitation was detected by qRT-PCR and agarose gel electrophoresis. The following primers were used:

- NOTCH1 promoter 1 Forward 5′-GGCTCCTCCGCTTATTCACAT-3′
  Reverse 5′-CGCCTGGGACTACTTCTCGT-3′.
- NOTCH1 promoter 2 Forward 5′-CTATGGCAGGCATTTTGGACT-3′
  Reverse 5′-GCTGATTTATTTCTCCACCACGA-3′.
- NOTCH1 promoter 3 Forward 5′-TAGGTCCCTCCCAGCCTTT-3′
  Reverse 5′-GCTGATTTATTTCTCCACCACGA-3′.

### Flow cytometry

Transfected cells were detached with trypsin and washed 1–2 times with cold phosphate-buffered saline (PBS). The cells were fixed with cool 70% ethanol at room temperature, and then washed again with PBS. The cells were immediately stained with propidium iodide using a BD Cycletest Plus DNA reagent kit (BD Biosciences, San Jose, CA, USA) following the manufacturer’s protocol. Analyses of cell cycle were performed using a FACS Calibur Flow Cytometer (Beckman Coulter, Atlanta, GA, USA).

### Establishment of macrophage, co-incubate and flow cytometry analysis

THP-1 cells were cultured in RPMI-1640 medium with 10% fetal bovine serum and 100 ng/ml Phorbol-12-myristate-13-acetate (PMA) for 72 h. The adherent THP-1 Cells induced by PMA were co-incubated with U87 cells stained with GFP fluorescence for 48 h. The THP-1 cells were then sorted and harvested by a SONY SH800 Cell Sorter. After washed by PBS twice, the sorted cells were incubated with Alexa Fluor® 647-conjugated anti-human CD206, Phycoerythrin-conjugated anti-human CD80, (all 1:100, Abcam). Multiple-color FACS analysis was performed using a FACS Calibur Flow Cytometer (Beckman Coulter, Atlanta, GA, USA) and analyzed by FlowJo software (TreeStar, San Carlos, CA).

### In vivo experiments

All experimental animal procedures were conducted strictly in accordance with the Guide for the Care and Use of Laboratory Animals and approved by the Animal Care and Use Committee of the Shandong provincial hospital affiliated to Shandong University. The male BALB/c nude mice were randomized divide into four groups in a blinded manner, each group including five 4-weeks-old nude mice. Two groups were used for subcutaneous xenograft study, and the other two groups were used for stereotactic intracranial tumor implantation.

For subcutaneous xenograft study, 5 × 10^5^cells were subcutaneously injected in the right flanks of nude mice. For stereotactic intracranial tumor implantation, 5 × 10^5^cells glioma cells were harvested by trypsinization, counted, and resuspended in culture medium. Mice were anesthetized by intraperitoneal administration of ketamine (132 mg/kg) and implanted using a stereotactic head frame at a depth of 3 mm through a bur hole placed 2 mm lateral and 0.5 mm anterior to the bregma. For histopathologic analysis, the mice brain were made into frozen sections with 8-μm thickness. Slides were incubated overnight at 4 °C with primary antibodies (anti-NKAP diluted at 1:100).

For study of tumor microenvironment, tissue was minced and digested with trypsin for 20 min at 37 °C. The homogenate was then filtered through a 40 μm filter and prepped using Fixation/Permeabilization solution according to the manufacturer’s instructions (BD Pharmingen. San Diego, CA). Cells were then incubated with FITC conjugated anti-mouse TMEM119 antibody, APC conjugated anti-mouse Gr-1 antibody, FITC conjugated anti-mouse Neutrophil (Ly-6B) antibody and FITC conjugated anti-mouse CD11b antibody prior to FACS analysis.

### Statistical analysis

Quantitative data were expressed as the mean ± standard deviation (SD). Significance was tested by one-way analysis of variance (ANOVA) or two-tailed t-tests among various groups. For in vivo studies, Kaplan-Meier curve and log-rank analyses were conducted using MedCalc software (Mariakerke, Belgium). *P* < 0.05 was considered statistically significant.

## Results

### NKAP affected the viability of glioma cells

**Fig. 1:**
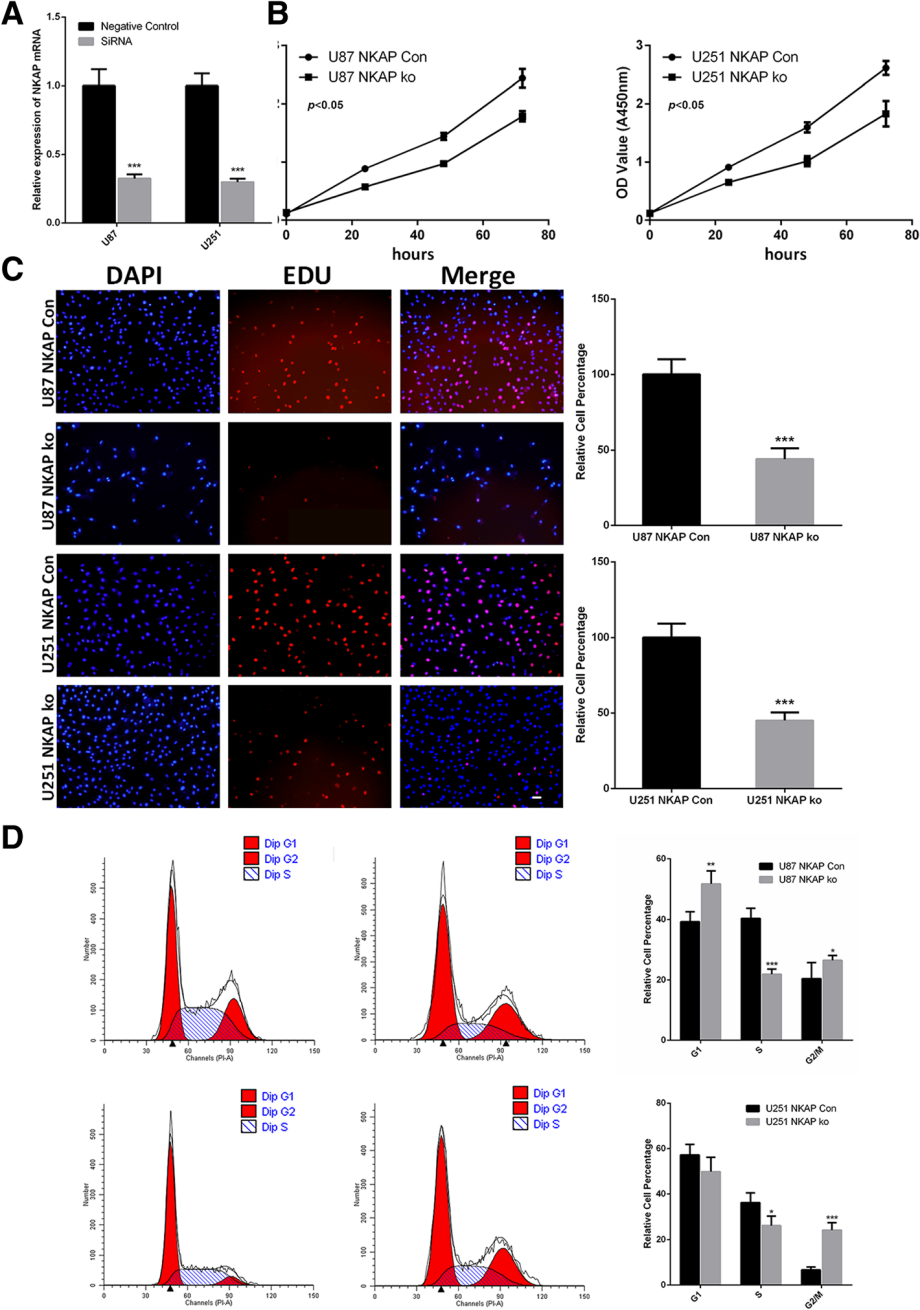
Effects of NKAP expression on the viability of glioma cells. U87 and U251 glioma cell lines were infected with lentiviruses expressing GFP and siRNA of NKAP. Nonspecific lentiviral vectors with nonspecific were used as negative controls. **a**. qRT-PCR assay was performed to test knockdown efficiency. **b**, The growth curves of the infected glioma cells were examined using a CCK8 assay. Cell growth was inhibited by NKAP knockdown. Data are presented as the mean ± s.d. of three independent experiments. **c**, An EdU staining assay was performed to test cell proliferation. The EDU staining (red) cells showed strong proliferative activity. Quantitative analysis showed that NKAP knockdown reduced the EdU incorporation rate. **d**, Cell populations in the G1, S and G2/M phases were analyzed by flow cytometry. The number of S phase cells were decreased following NKAP knockdown in both cell lines. Scale bar = 20 μm. The results represent the mean ± s.d. of three independent experiments. ***P < 0.001, **P < 0.01,*P < 0.05

To elucidate the functions of NKAP in gliomas, we firstly tested the effects of NKAP on glioma cell growth. We infected both U87 and U251 glioma cells with the lentiviruses expressing GFP and siRNA of NKAP. Nonspecific lentiviral vectors were used as the negative control. qRT-PCR and western blot analysis indicated a decrease by approximately 70% in the si-NKAP-infected cells compared with the scrambled siRNA-infected cells (Fig 1a, Additional file 1: Figure S1).

An CCK8 cell viability assay was applied to evaluate whether NKAP could affect the viability of glioma cells. As depicted in Fig. 1b, depletion of NKAP resulted in a significant inhibition of U87 and U251 cell proliferation. The EDU assay results also supported this phenomenon (Fig. 1c). In addition, cell cycle flow cytometry showed that depletion of NKAP resulted in a marked inhibition of the S phase among glioma cells (Fig. 1d). The S phase decreased in both two cell lines which indicated proliferation inhibition, but changes of G1 phase were different. These would suggest that the proliferation of two cell lines were both inhibited, but the cell cycle progressions were stuck in different phases. Li et al. has reported that U87 cells exhibited a greater capacity for proliferation and invasion than U251 cell. In addition, by using gene chip analysis, they found that different biological functions existed between the U87 and U251 cell lines [23]. These may explain this interesting phenomenon. Collectively, these data suggested that NKAP was indeed involved in the proliferative ability of gliomas by inducing G1/S arrest, especially in U87 cells.

### NKAP promoted the migration, invasion and EMT of glioma cells

**Fig. 2:**
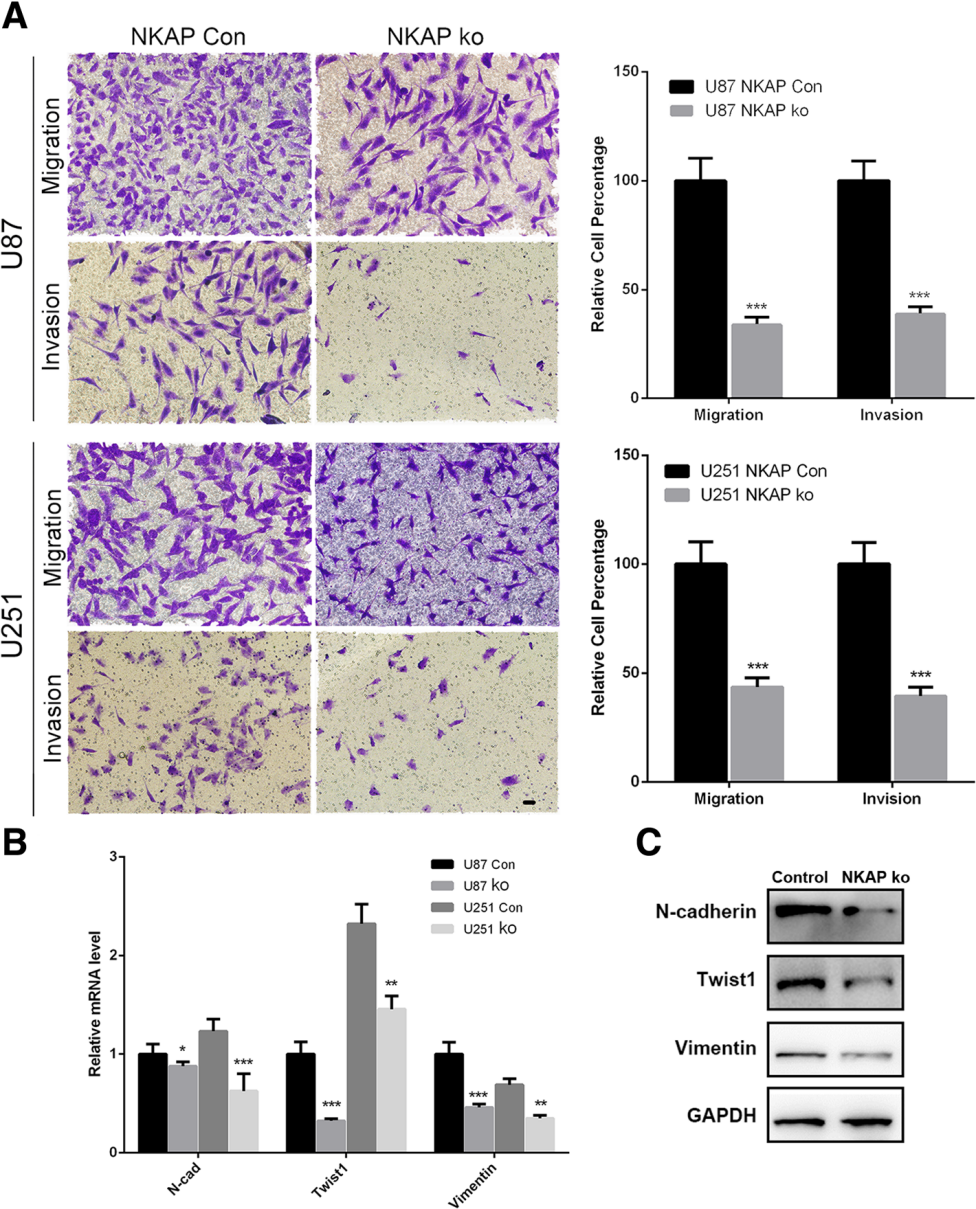
Effects of NKAP on the migration, invasion and EMT of glioma cells. **a**, Cell migration and invasion were determined by a transwell assay. Quantitative analysis showed that NKAP knockdown reduced glioma cell migration and invasion in different cell lines. ***P < 0.005 vs. Con. Levels of the representative EMT markers including N-cadherin, Twist1 and Vimentin were measured by qRT-PCR (**b**) and western blot (**c**) respectively. GAPDH was used as an internal control. Scale bar = 20 μm. The results represent the mean ± s.d. of three independent experiments. ***P < 0.001, **P < 0.01, *P < 0.05

To further investigate whether NKAP was related to glioma cell migration and invasion, we used a transwell assay to examine the effects of NKAP on U87 and U251 cell movement. As shown in Fig. 2a, NKAP knockdown led to significantly fewer migrating or invading cells than transfection with a scrambled vector (Fig. 2a). Epithelial-to-mesenchymal transition (EMT) is regarded as the key step, which particularly involves changes in cell-cell and cell-matrix interactions [24, 25]. To verify whether NKAP participated in EMT of glioma cells, we detected the mRNA levels of several representative EMT markers. As the results shown in Fig. 2b, the expression levels of mesenchymal markers such as N-cadherin, Twist1 and Vimentin were significantly decreased in both U87 and U251 cells knockdown with NKAP (Fig. 2b). Immunoblotting also confirmed this phenomenon (Fig. 2c), evidently suggesting the important role of NKAP in glioma cell migration and invasion.

### Knockdown of NKAP attenuated the growth of gliomas in vivo

**Fig. 3:**
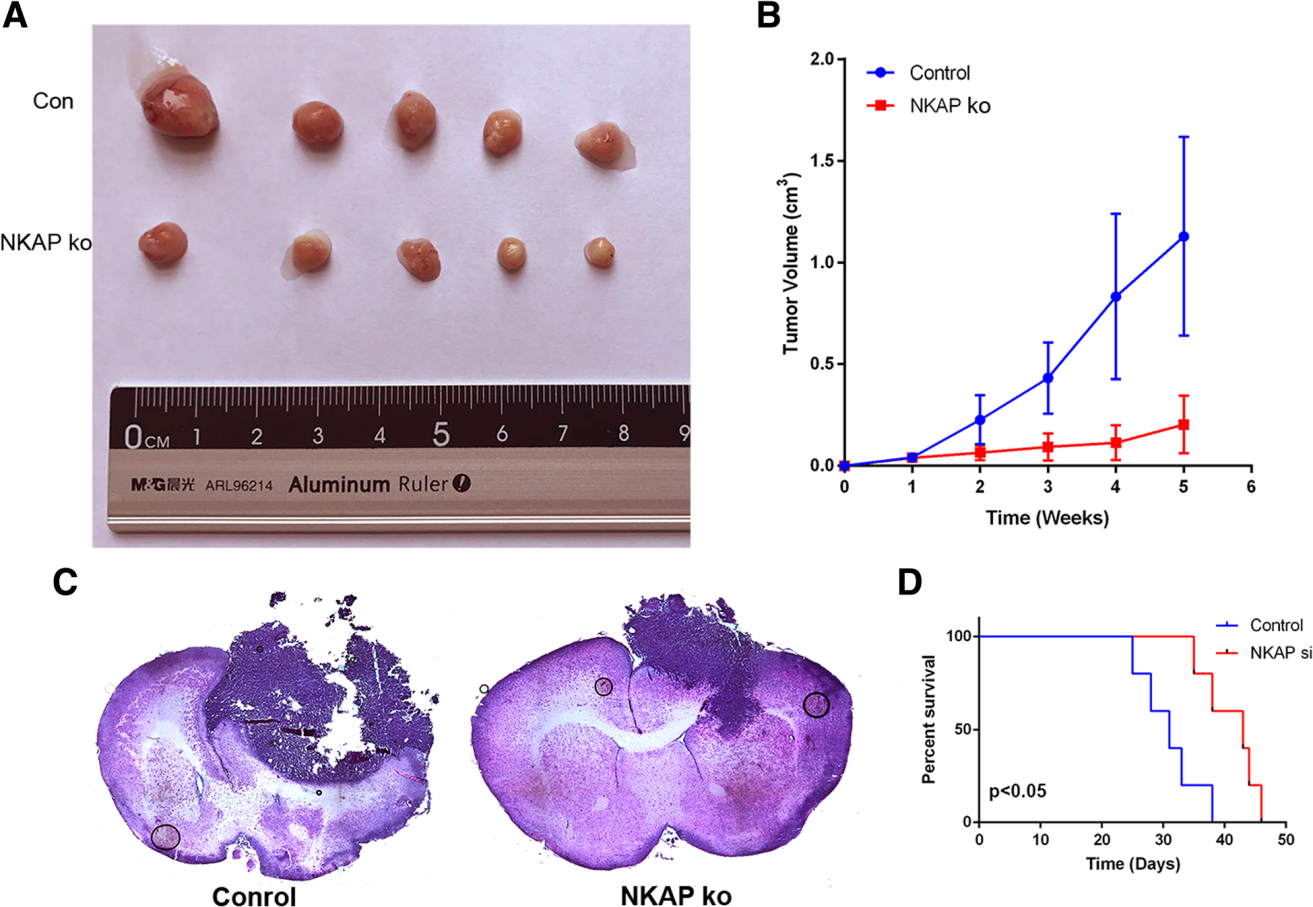
Knockdown of NKAP attenuated growth of gliomas in combination with down-regulation of Notch1 in vivo. The U87 cells transduced with a lentiviral vector expressing si-NKAP were subcutaneous and orthotopic implanted into immunocompromised mice respectively. *N* = 5 in each group. **a**, Representative xenograft tumors at 35 days after inoculation. **b**, The line chart shows the estimated tumor volumes at the indicated time. **c**, Representative micrographs of H&E-stained sections of mouse brain tissue 35 days after intracranial implantation of U87 cells transfected with NKAP-siRNA or control vectors. **d**, Curves show the survival rates of the engrafted mice

Considering the in vitro involvement of NKAP in glioma cell proliferation, invasion, migration and EMT, we extended this study to determine the impact of NKAP on tumorigenic capabilities of gliomas in vivo. When the U87 cells transduced with lentiviral vectors expressing NKAP-targeting siRNA or non-targeting control siRNA were subcutaneously implanted into the immunocompromised mice, we observed a significant decrease in tumor formation in the tumor-bearing mice when NKAP was inhibited (Fig. 3a and b). Similar results were manifested in the intracranially injected mice (Fig. 3c), which achieved longer survival when NKAP was down-regulated (Fig. 3d).

### NKAP altered recruitment and polarization of tumor-associated macrophages via regulating the secretion of SDF-1 and M-CSF

**Fig. 4:**
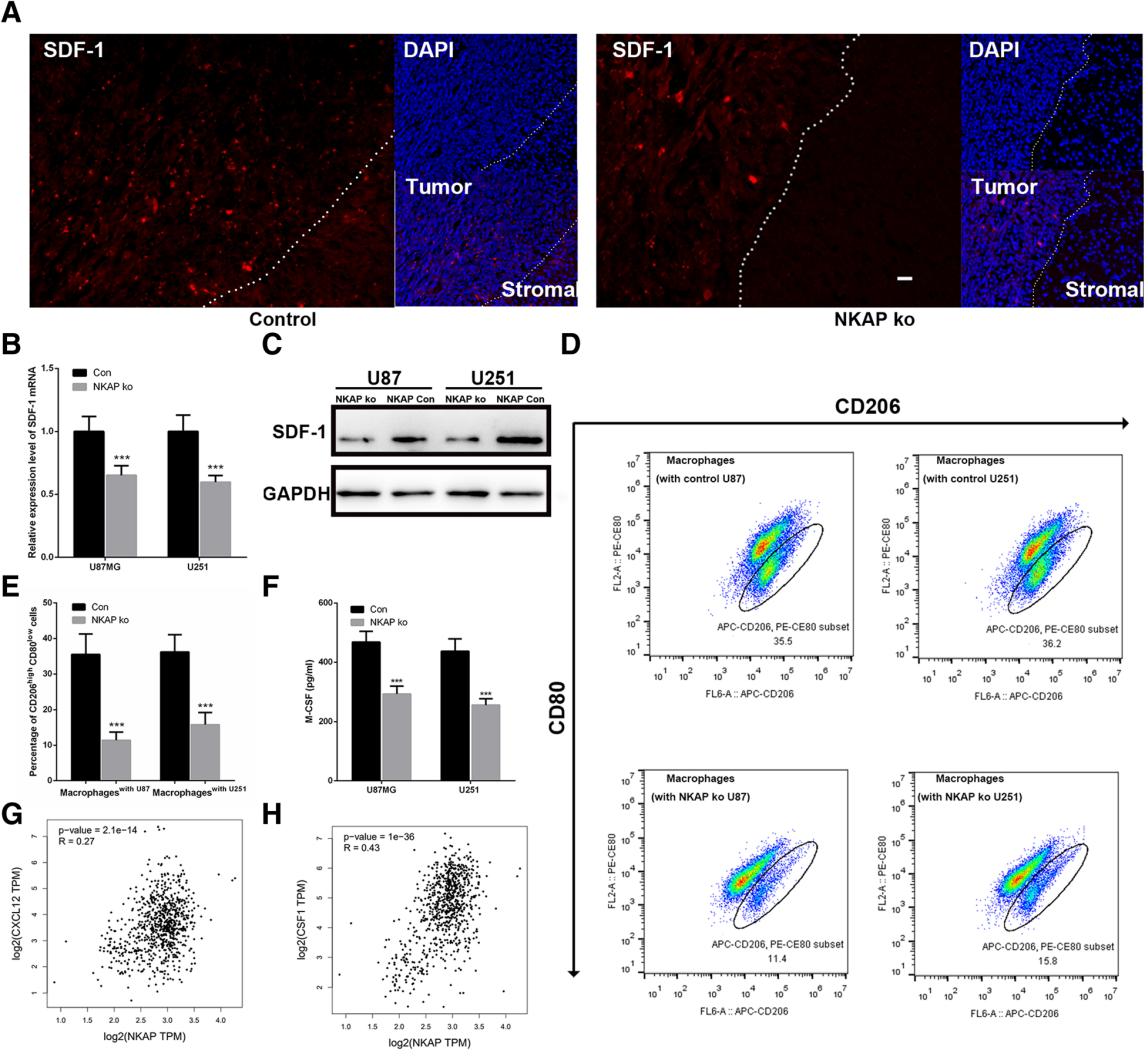
NKAP altered recruitment and polarization of TAMs via regulating the secretion of SDF-1 and M-CSF. **a**, Representative micrographs of mouse brain sections 35 days after intracranial implantation of U87 cells transfected with NKAP-siRNA or control vectors. Expression of SDF-1 was inhibited in NKAP knockdown glioma tissues. Levels of SDF-1 were measured by qRT-PCR (**b**) and western blot (**c**) in the cells transfected with NKAP-siRNA or control vectors. FACS quantification (**d**) and representative bar plots (**e**) demonstrated a decrease in the proportion of TAMs (i.e. CD206^high^, CD80^low^) following a co-incubation with NKAP knockdown U87 and U251 cells. **f**, Knockdown NKAP reduced the secretion of M-CSF in the glioma cells. **g** and **h**, Analysis of TCGA database about glioma tissues (*n* = 370) shows significant correlation between expressions of NKAP and M-CSF (CSF1)/SDF-1 (CXCL12). Scale bar = 20 μm. Data are presented as the mean ± s.d. of three independent experiments. ***P < 0.001

It was previously reported that NAKP was closely related to activation of the immune system. We therefore evaluated its effects on tumor inflammatory responses. The stromal cell-derived factor 1 (SDF1), also known as C-X-C motif chemokine 12 (CXCL12), has been implicated in the recruitment of monocytes/macrophages to the bulk of tumors. Macrophage colony-stimulating factor (M-CSF), on the other hand, is a secreted cytokine that causes macrophages to differentiate into tumor-associated macrophages (TAM) by binding to the colony stimulating factor 1 receptor (CSF1R). When we looked at the tumor-stromal boundary in the xenografted mice, a decrease in SDF-1 expression was observed in the glioma tissues knockdown of NKAP (Fig. 4a). Consistently, the mRNA and protein levels of SDF-1 were also down-regulated in the NKAP depleted glioma cells (Fig. 4b, c). We subsequently co-cultured the glioma cells with macrophages (THP-1 cell induced by RMA). The proportion of CD206^high^ macrophages (TAMs) co-cultured with NKAP knockdown U87 and U251 cells was much less than those co-cultured with control cells, suggesting that NKAP was involved in the altered polarization of TAMs (Fig. 4d, e). We additionally detected the cytokines released by the co-cultured glioma cells, and found that NKAP positively controlled M-CSF secretion (Fig. 4f). To elucidate the correlations between NKAP and SDF1/M-CSF, we took a step further to analyze the TCGA database. The results showed that NKAP was indeed positively correlated with SDF1 (R = 0.27, *p* < 0.001) and M-CSF (R = 0.43, *p* < 0.001) (Fig. 4g, h). Collectively, these results evidently suggested that NKAP was involved in the recruitment and polarization of TAMs in gliomas.

### Notch1 was an NKAP target gene

**Fig. 5:**
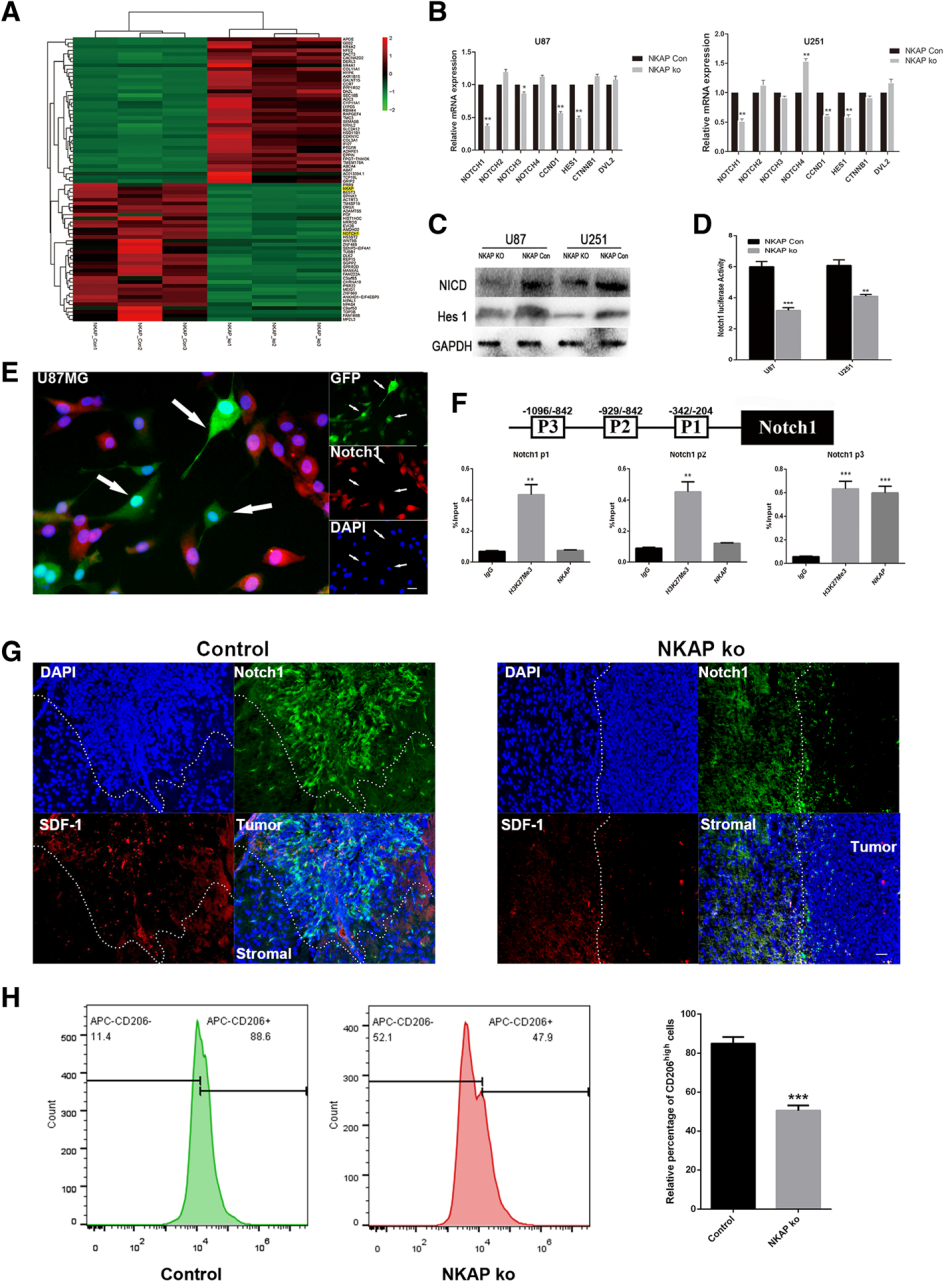
Notch1 is a potential NKAP target gene associated with the TME of glioma. **a**, Gene expression profile of control and NKAP knockdown U87 cells. Red: High level expression; Green: Low level expression. **b**, mRNA expression levels of Notch family and Notch signaling pathway genes were determined by qRT-PCR. Data are presented as the mean ± s.d. of three independent experiments. *P < 0.05, **P < 0.01, ***P < 0.005 vs. Con. **c**, The protein levels of NICD1 and HES1 were analyzed by western blot in the NKAP knockdown cell lines. GAPDH serves as a loading control. **d**, The full length human Notch1 promoter (− 2000 bp to + 0 bp) was cloned into the luciferase reporter vector. Notch1 transcription activity was examined after down-regulating the expression of NKAP in U87 and U251 cell lines. **e**, The lentiviruses carried GFP gene, so the NKAP knockdown cells showed green fluorescence. Notch1 (red) expression was significantly decreased in the NKAP knockdown U87 cells (green). **f**, ChIP analysis was performed by using antibody against NKAP with primers targeted to the promoter region of Notch1. Isotype-matched IgG was used as a negative control. PRL30-matched H3 was used as a positive control. **g**, Notch1 and SDF1 expressions were significantly decreased in the NKAP knockdown glioma tissues. **h**, Downregulation of NKAP reduced the proportion of CD206^high^ macrophages in the mouse brain glioma tissues. Scale bar = 20 μm. Data are presented as the mean ± s.d. of three independent experiments. ***P < 0.001, **P < 0.01

To further identify the potential NKAP targets in gliomas, we performed RNA sequencing in triplicates to determine the gene expression profiles of the control and NKAP knockdown cell lines (Fig. 5a). Interestingly, Notch1 was one of the most significantly down-regulated genes (2.7-fold decreased expression) that was correlated with NKAP depletion. We additionally performed GO and KEGG analysis on the differential expression profiles. The results of GO analysis showed that NKAP was significant associated with cytokine production involved in the immune response. The KEGG analysis, on the other hand, indicated that NKAP was indeed involved in regulation of Notch signaling pathway (Additional file 2: Figure S2). It is well established that Notch1 signaling plays a critical role in various human cancers including gliomas. It was also reported to transactivate and induce the secretion of SDF-1 and M-CSF, contributing to an immuno-suppressive tumor microenvironment. As such, we speculated that Notch1 might be the potential target of NKAP in the regulation of glioma development and progression.

To verify this hypothesis, qRT-PCR and western blot were performed in the U251 and U87 cell lines to assess the expressions of the genes within Notch signaling pathway. The results showed that depletion of NKAP significantly inhibited both the mRNA and protein expression levels of Notch1, NICD and Hes1. In contrast, the levels of Notch2, Notch3 and Notch4 were moderately regulated (Fig. 5b, c). The above results were further confirmed by the luciferase assay by revealing that down-regulation of NKAP reduced Notch1 luciferase activity (Fig. 5d). When we examined the level of Notch1 with immunofluorescence analysis, we could clearly detect significant loss of Notch1 expression in the cells transfected with sh-NKAP in comparison to the control (Fig. 5e). We next questioned the specific mechanism of NKAP in Notch1 trans-activation. A chromatin immunoprecipitation (ChIP) assay was conducted in the U87 cells to detect the different regions of Notch1 promoter that could potentially bind to NKAP. Interestingly, a significant enrichment of endogenous NKAP protein was detected at the Notch1 promoter region 3 (Fig. 5f, Additional file 3: Figure S3). As such, we could surmise that NKAP may function in glioma cells via directly binding to the Notch1 promoter.

When we performed an immunohistological analysis on the paraffin-embedded brain tumor samples in the xenografted mice. In contrast to a higher immunostaining of Notch1 and SDF-1 in the gliomas derived from the cells transduced with scrambled control shRNA, the gliomas depleted of NKAP displayed much lower levels of these two factors, evidently suggesting a regulatory role of NKAP in Notch1 signaling (Fig. 5g). We took a step further to analyze various immune cell components in the mouse brain tumors. The results showed that proportion of CD206^high^ macrophages was much less in the gliomas knockdown with NKAP in comparison to scrambled controls (Fig.5h). In addition, percentage of myeloid-derived suppressor cells (CD11b^+^, Gr-1^+^) and neutrophils (Ly6B^+^) were significantly down-regulated (Additional file 4: Figure S4A-S4B). Considering that CD206 is expressed in both macrophages and microglia, we stained TMEM119, a specific marker for microglia to determine whether NKAP recruited immune suppressive macrophages or reprogrammed microglia. The results showed that the proportion of TMEM119 positive microglia was not affected by NKAP depletion (Additional file 4: Figure S4C). Taken together, these results evidently suggested that NKAP together with Notch1 signaling was involved in regulation of the immune microenvironment of gliomas.

### Relevance between the expressions of NKAP and Notch1 in clinical samples

**Fig. 6:**
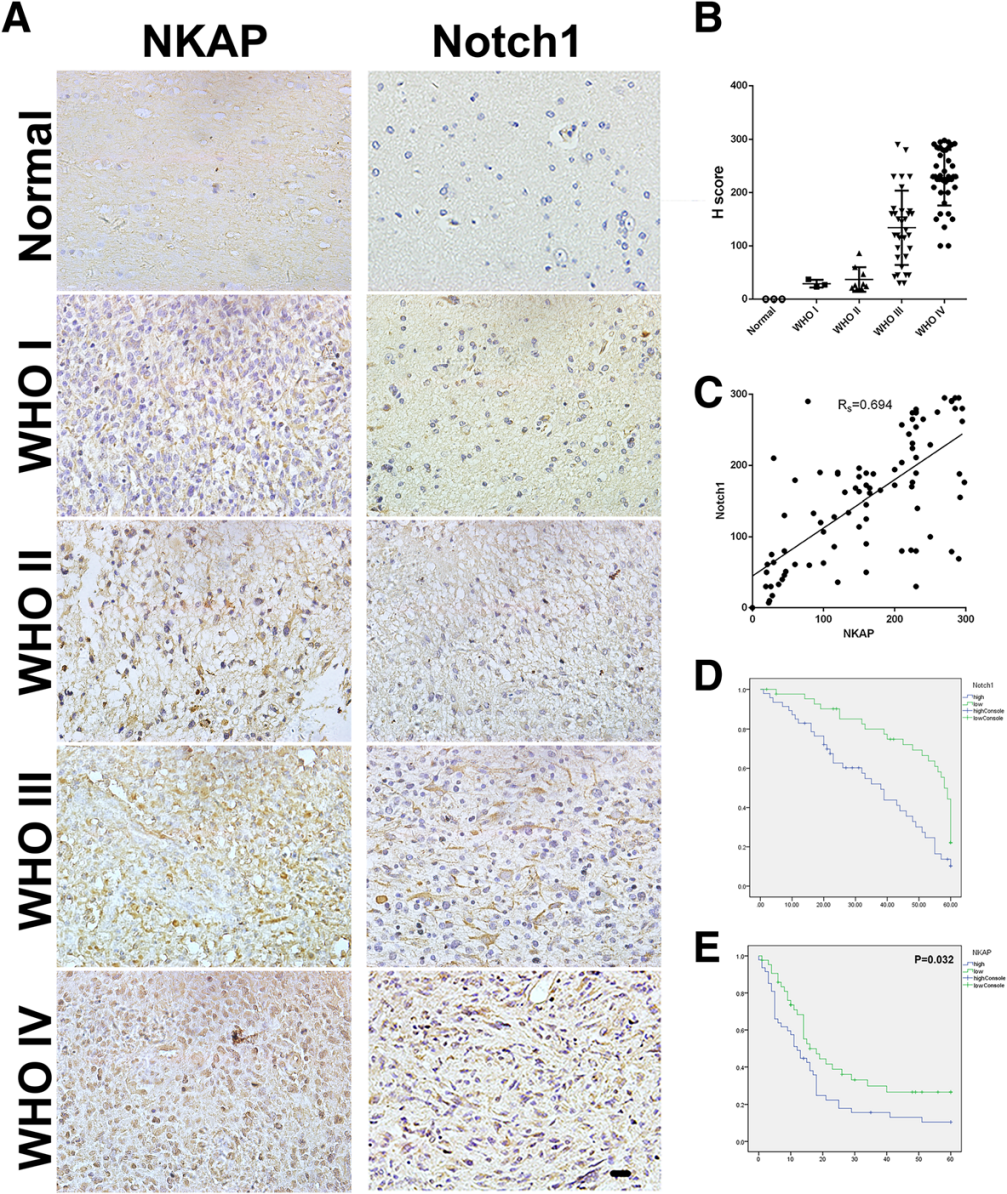
Correlated NKAP and Notch1 expression in glioma tissues. **a**, IHC was performed to detect NKAP and Notch1 expression in normal brain specimens and in grade I, II, III, and IV glioma specimens (400×). **b**, The intensity of Notch1 expression in glioma specimens significantly increased as tumor malignancy increased. **c**, A remarkable positive correlation between the expression of NKAP and Notch1 was observed in 90 glioma specimens (*P* < 0.01). **d** and **e**, The overall survival of the 90 glioma patients was assessed based on low or high expression levels of NKAP and Notch1. The higher expression of NKAP and Notch1 indicated a worse prognosis. Scale bar = 20 μm

To further strengthen the evidence that Notch1 expression is correlated with NKAP in the development of gliomas, immunohistochemical analysis was conducted to examine the immunostaining of both Notch1 and NKAP in the tissue array of 90 patient samples comprising normal brain tissues as well as glioma tissues classified into grade I, II, III, and IV. Similar to NKAP, the expression intensity of Notch1 in the tissues was correlated with degrees of malignancy (Fig. 6a, b). Furthermore, a remarkable positive correlation between these two proteins were shown, evidently supporting Notch1 as a potential target of NKAP in human gliomas (Fig. 6c). To assess the epidemiological value of NKAP in glioma patients, the 90 patients with gliomas from different pathological grades were divided into two groups based on the relative expression levels of NKAP. The cases in which transcripts were elevated to a level above the median were classified as the high-level NKAP group. The remaining patients were classified as the low-level NKAP group. During the five-year follow-up period, the overall survival of the glioma patients with high-level NKAP expression was markedly lower than that of patients with low-level NKAP expression (Fig. 6d), suggesting that a high level of NKAP was associated with poor prognosis in a long round. Very similarly, the overall survival of the glioma patients with high-level of Notch1 expression was markedly lower than that of patients with low-level of Notch1 expression (Fig. 6e), consistent with the report of Engh JA [26] who previously implicated Notch1 as a prognostic factor for glioma patients. These data strongly suggested that NKAP could be regarded as a significant predictor of glioma prognosis in the overall population. Additionally, it is highly possible that the effects of NKAP in gliomagenesis was mediated by Notch1.

### Blockage of Notch1 alleviated the effects of NKAP in gliomas

**Fig. 7:**
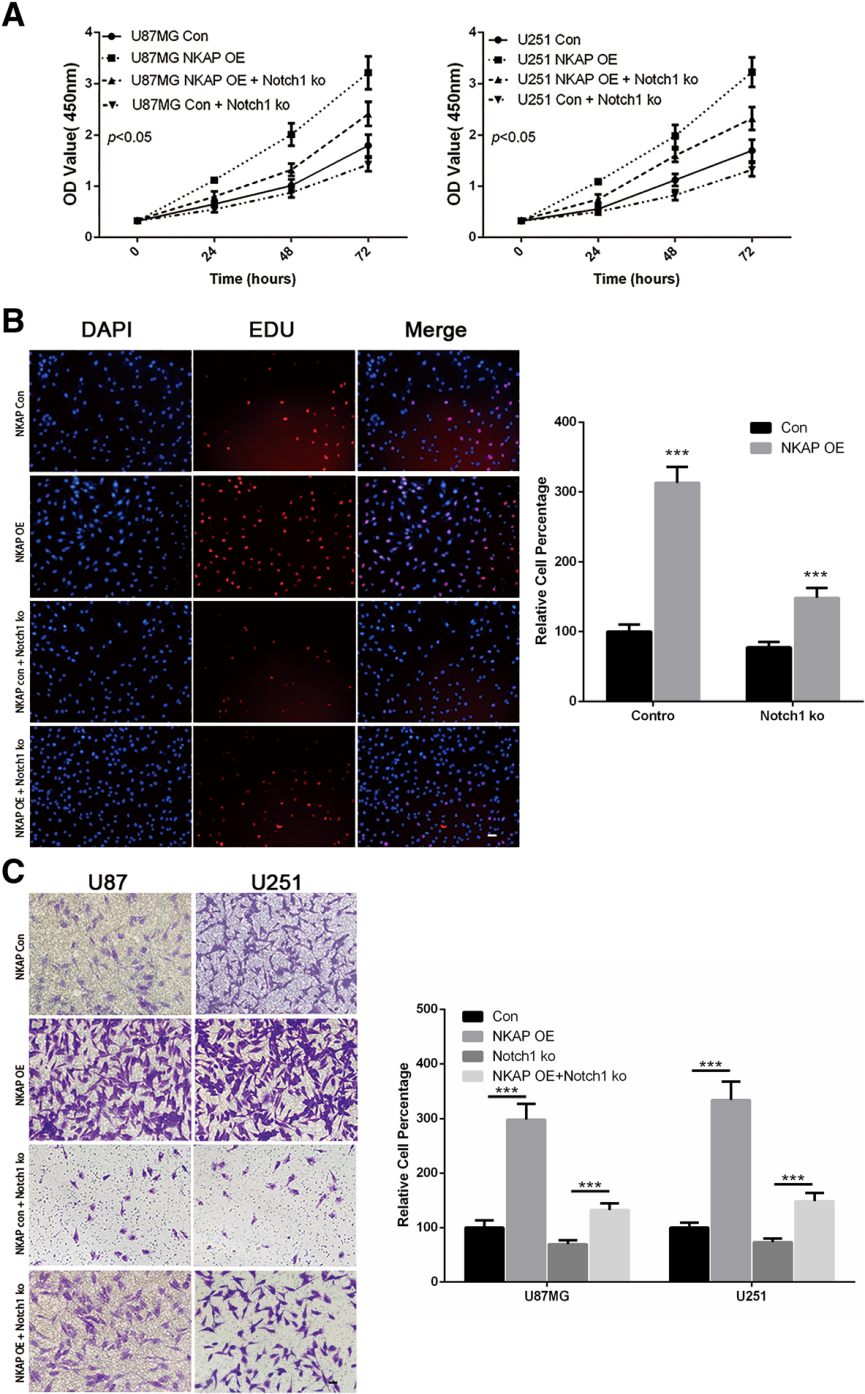
Blockage of Notch1 weakened the effects of NKAP on the proliferation and invasion of glioma cells. The U87 and U251 cell lines were transfected with NKAP plasmids to overexpress NKAP, which were termed NKAP OE cells. DAPT was also applied to the transfected cell lines. **a**, The growth curves of the transfected cells plotted based on a CCK8 assay. After inhibiting Notch1 signaling by DAPT, the cell viability was decreased compared with that of NKAP OE cells. Data are presented as the mean ± s.d. of three independent experiments. **b**, Cell proliferation was determined by an EdU staining assay. **c**, Cell invasion was determined by a transwell assay. The results represent the mean ± s.d. of three independent experiments. Scale bar = 20 μm. The results represent the mean ± s.d. of three independent experiments. ***P < 0.001

**Fig. 8:**
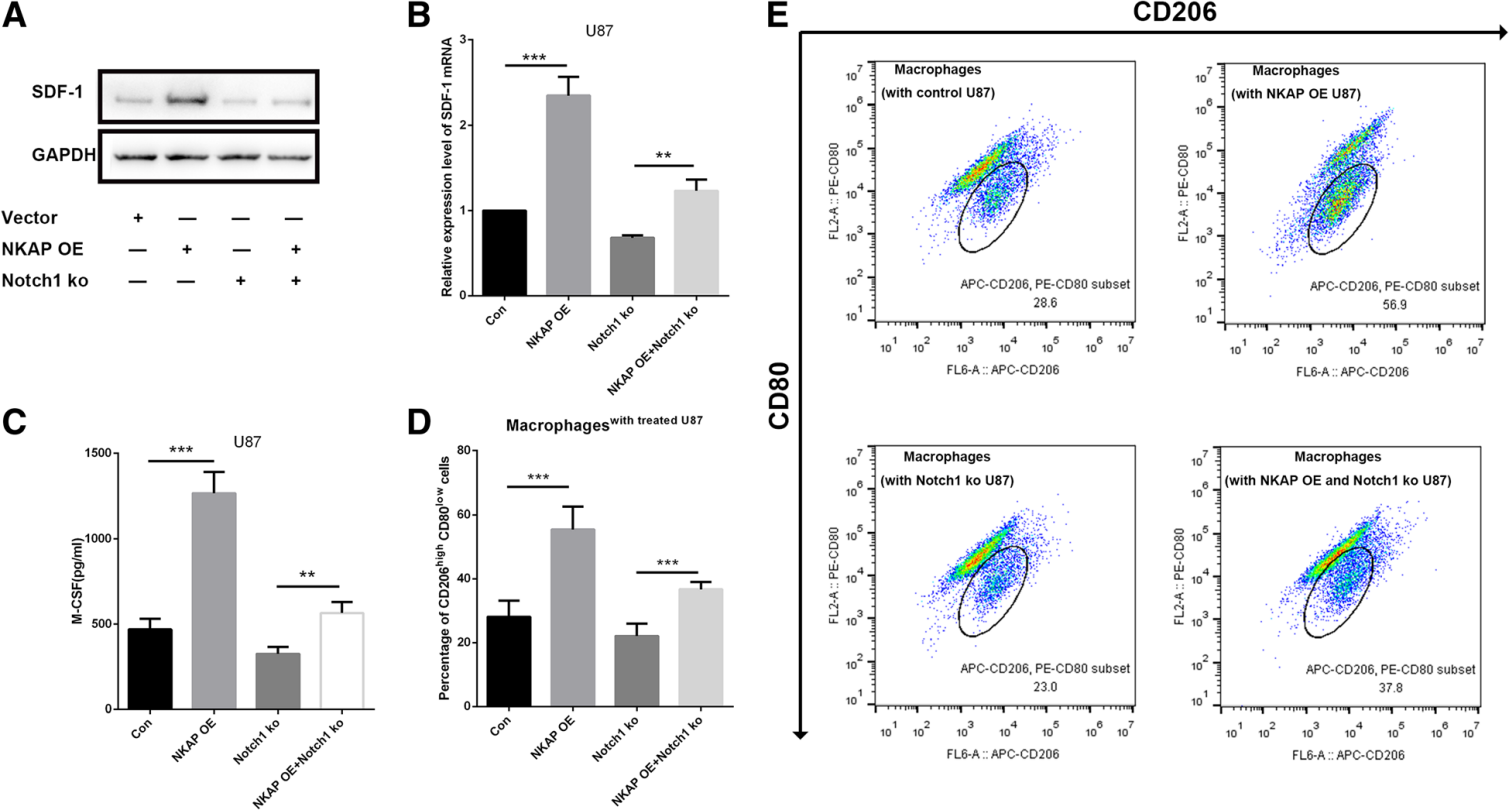
Blockage of Notch1 attenuated the effects of NKAP on the TME of glioma. **a** and **b**, Inhibiting Notch1 signaling reduced the function of NKAP in promoting SDF-1 expression. **c**, Downregulation of Notch1 signaling weakened the function of NKAP in promoting M-CSF secretion. **d**, After inhibiting Notch1 signaling in U87 cells, the increased proportion of TAMs (CD206^high^ CD80^low^) which co-incubated with NKAP overexpressed U87 cells was decreased compared to control group. Data are presented as the mean ± s.d. of three independent experiments. ***P < 0.001, **P < 0.01

Given that NKAP promotes glioma cell proliferation and invasion and that Notch1 is a potential target of NKAP, we next investigated whether Notch1 represented a functional link for the biological changes observed in the glioma cells with NKAP deletion. To confirm this hypothesis, U87 and U251 cell lines were first transfected with the plasmids overexpressing NKAP, which were termed NKAP OE cells. Furthermore, we transfected these NKAP OE cells with sh-RNA designated to Notch1 to investigate the responses to Notch1 inhibition. As expected, the efficiency of NKAP in promoting cell viability (Fig. 7a), proliferation (Fig. 7b) and invasion (Fig. 7c) were significantly decreased when Notch1 was inhibited. Moreover, Downregulation of Notch1 significantly decreased the elevated secretion of SDF-1 and M-CSF in NKAP OE U87 cells (Fig. 8a-c) and attenuated their effects in TAM polarization (Fig. 8d, e).

## Discussion

Glioma is among the deadliest type of brain tumors with a high degree of malignancy and poor prognosis. With in-depth study, the brain tumor microenvironment (TME) has emerged as a critical regulator of glioma progression, within which immune tolerance and suppression are the key regulatory factors. The TME contains many different non-cancerous cell types in addition to cancer cells, such as endothelial cells, pericytes, fibroblasts and immune cells. Among them, the majority of immune cells are macrophages, often comprising up to ~ 30% of the tumor mass [27]. However, whether these cells have distinct functions in the brain TME has been controversial, and remains as a topic of active investigation for a long time. Clarifying the molecular mechanisms beneath would provide a theoretical basis for developing effective treatment strategies or identifying new therapeutic targets.

NKAP plays an important role in neural development considering its interesting expression patterns in the neural system. It was reported that NKAP is expressed at heterogeneous levels in different parts of the brain, with a higher expression in the proliferative progenitor cell types in the SVZ region, but a lower expression in the adult neural cells such as glial cells [12]. These results evidently suggested that NKAP might be involved in the regulation of neural stem or progenitor cell identity. Since increased expression of stemness related genes are usually pronounced in the malignancy, it has drawn our great attention how NKAP is expressed in the glial-derived tumor cells.

By use of tissue collection and immunohistochemical analysis, we observed that the expression of NKAP was significantly upregulated in the gliomas. The level of increase in NKAP expression was positively correlated with the degree of glioma malignancy and inversely correlated with the prognosis. More importantly, we detected that cellular proliferation, migration and invasion were significantly inhibited upon NKAP knockdown in the cell lines of gliomas. Furthermore, downregulation of NKAP could reduced the recruitment and polarization of TAM by decreasing the secretion of SDF-1 and M-CSF. As a corollary, NKAP seemed to be a key regulator of glioma progression and TME, but its molecular mechanisms still remain unclear.

In order to explore the mechanisms of NKAP in gliomagenesis, we performed RNA sequencing analysis to determine differentially expressed genes affected by NKAP. Notch1 was observed as one of the most closely related genes. The regulatory relationship between NKAP and Notch1 was firstly reported in mammalian T cells. Pajerowski has reported that NKAP could directly interact and co-localize with the known Notch co-repressors CIR and HDAC3 in the regulation of mammalian T-cell development, resulting in suppression of Notch target genes [7]. Nevertheless, the concrete mechanisms of NKAP in the regulation of Notch1 signaling has not been thoroughly investigated in details, especially in brain cells and tumors. In this study, in contrast to the result derived from T-cell development, we observed that down-regulation of NKAP inhibited the expression of Notch1 in vivo and in vitro*.* Instead of a repressor component, NKAP transactivated Notch1 in the glioma cells. To make a step further, we carried out a ChIP assay and detected a direct binding between NKAP and Notch1 promoter region. Notch1 inhibition could indeed alleviated the functions resulted from upregulation of NKAP. Overall, our findings provided new insight into the regulatory relationship between NKAP and Notch1 in the tumorigenesis of gliomas.

In the nervous system, mounting evidences have revealed that aberrant Notch signaling has been closely involved in the development of gliomas. Among them, a critical role of Notch1 in regulation of immune suppressive TME has drawn active attention. According to Ling’s study, activating Notch1 signaling could promote the expression of M-CSF in BV2 cells [28], though there was another opposite conclusion reported by Sakano that constitutively active Notch1-transfected stromal cells showed strong inhibition of M-CSF gene expression [29]. On the other hand, Yang has confirmed that overexpression of notch1 augmented SDF-1-induced chemotaxis in cancer stem cells in vitro [30]. Based on our data that NAKP controlled the expression and secretion of SDF-1 and M-CSF via Notch1, it could be concluded NKAP was involved in regulation of the tumor immune microenvironment of gliomas. As a corollary, we have demonstrated a model where NKAP positively regulates the expression of Notch1, which results in an increasing secretion of M-CSF of SDF-1 in glioma cells [31], reinforcing significant bidirectional cross-talks between macrophages and glioma cells. M-CSF plays an important role in transforming macrophages into TAMs, while SDF-1 promotes their recruitment. These TAMs in turn secret various pro-tumorigenic factors, such as TGF-β, which ultimately promote the growth of glioma. This model evidently suggested a critical role of NKAP in the feedback loop between glioma development and tumor immune microenvironment (Additional file 5: Figure S5).

In summary, we have identified NKAP as an important oncogenic factor in gliomas, and indicated its ability to promote glioma proliferation and invasion. What’s more, we provided unequivocal evidence for the first time demonstrating that NKAP performed its function in part via regulating glioma immune microenvironment through targeting Notch1. These novel findings would provide a new perspective for glioma chemotherapeutic intervention.

## Conclusion

In this manuscript, we have identified NKAP as an important oncogenic factor in gliomas. What’s more, we provided unequivocal evidence for the first time demonstrating that NKAP performed its function in part via regulating glioma immune microenvironment through targeting Notch1.

## Additional files

Additional file 1: **Figure S1.** Western blot assay was performed to test the knockdown efficiency of NKAP in U87, U251 and GL261 cells. (TIF 906 kb)

Additional file 2: **Figure S2.** GO and KEGG analyses were based on the RNA sequencing profiles resulted from NKAP knockdown. Both cytokine production involved in the immune response and Notch signaling pathway were significantly affected. (TIF 3542 kb)

Additional file 3: **Figure S3.** Agarose gel electrophoresis of the CHIP assay. It was performed by using antibody against NKAP with primers targeted to the promoter region of Notch1. Isotype-matched IgG was used as a negative control. (TIF 695 kb)

Additional file 4: **Figure S4.** Proportion of myeloid-derived suppressor cells (A) and neutrophils (B) were significantly down-regulated in the NKAP depleted gliomas. C, Percentage of TMEM119 positive microglia was not affected by NKAP knockdown. (TIF 560 kb)

Additional file 5: **Figure S5.** Mechanism map of NKAP in the feedback loop between glioma development and tumor immune microenvironment. (PNG 169 kb)

## Data Availability

All the data and materials are available.

## Abbreviations

CSL: CBF-1, Suppressor of hairless, Lag
HDAC: Histone deacetylase 3
iNKT: invariant natural killer T
M-CSF: Macrophage colony-stimulating factor
NCI: National Cancer Institute
NICD: Notch intracellular domain
NKAP: NF-κB activating protein
NSC: neural stem cell
SDF-1: Stromal cell-derived factor 1
SVZ: subventricular zone
TAMs: Tumor-associated macrophages
TME: Tumor microenvironment
