# Supplementary figures and images for: NKAP alters tumor immune microenvironment and promotes glioma growth via Notch1 signaling
Source: J Exp Clin Cancer Res. 2019 Jul 6;38:291. doi: 10.1186/s13046-019-1281-1 (PMC6612223; doi:10.1186/s13046-019-1281-1)

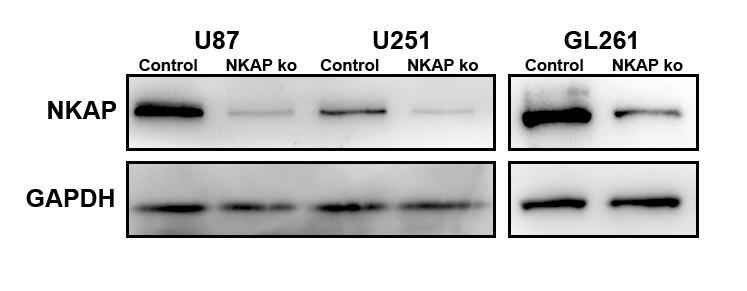

Supplement: Supplementary file 1 — Figure S1. Western blot assay was performed to test the knockdown efficiency of NKAP in U87, U251 and GL261 cells. (TIF 906 kb) [file 13046_2019_1281_MOESM1_ESM.tif]

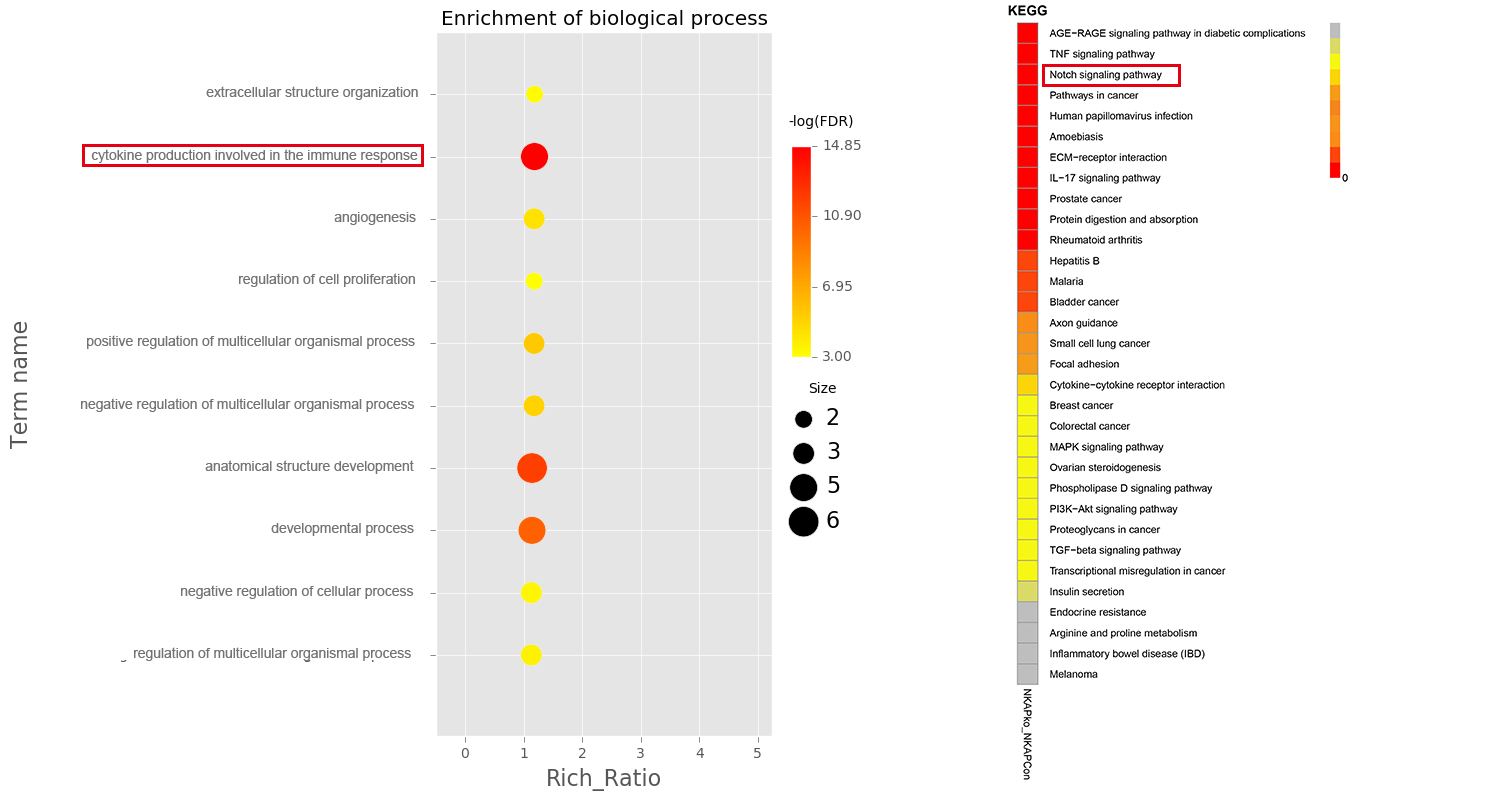

Supplement: Supplementary file 2 — Figure S2. GO and KEGG analyses were based on the RNA sequencing profiles resulted from NKAP knockdown. Both cytokine production involved in the immune response and Notch signaling pathway were significantly affected. (TIF 3542 kb) [file 13046_2019_1281_MOESM2_ESM.tif]

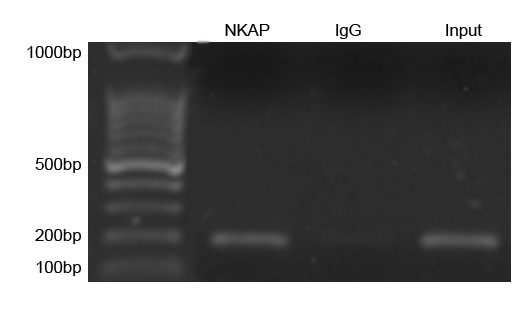

Supplement: Supplementary file 3 — Figure S3. Agarose gel electrophoresis of the CHIP assay. It was performed by using antibody against NKAP with primers targeted to the promoter region of Notch1. Isotype-matched IgG was used as a negative control. (TIF 695 kb) [file 13046_2019_1281_MOESM3_ESM.tif]

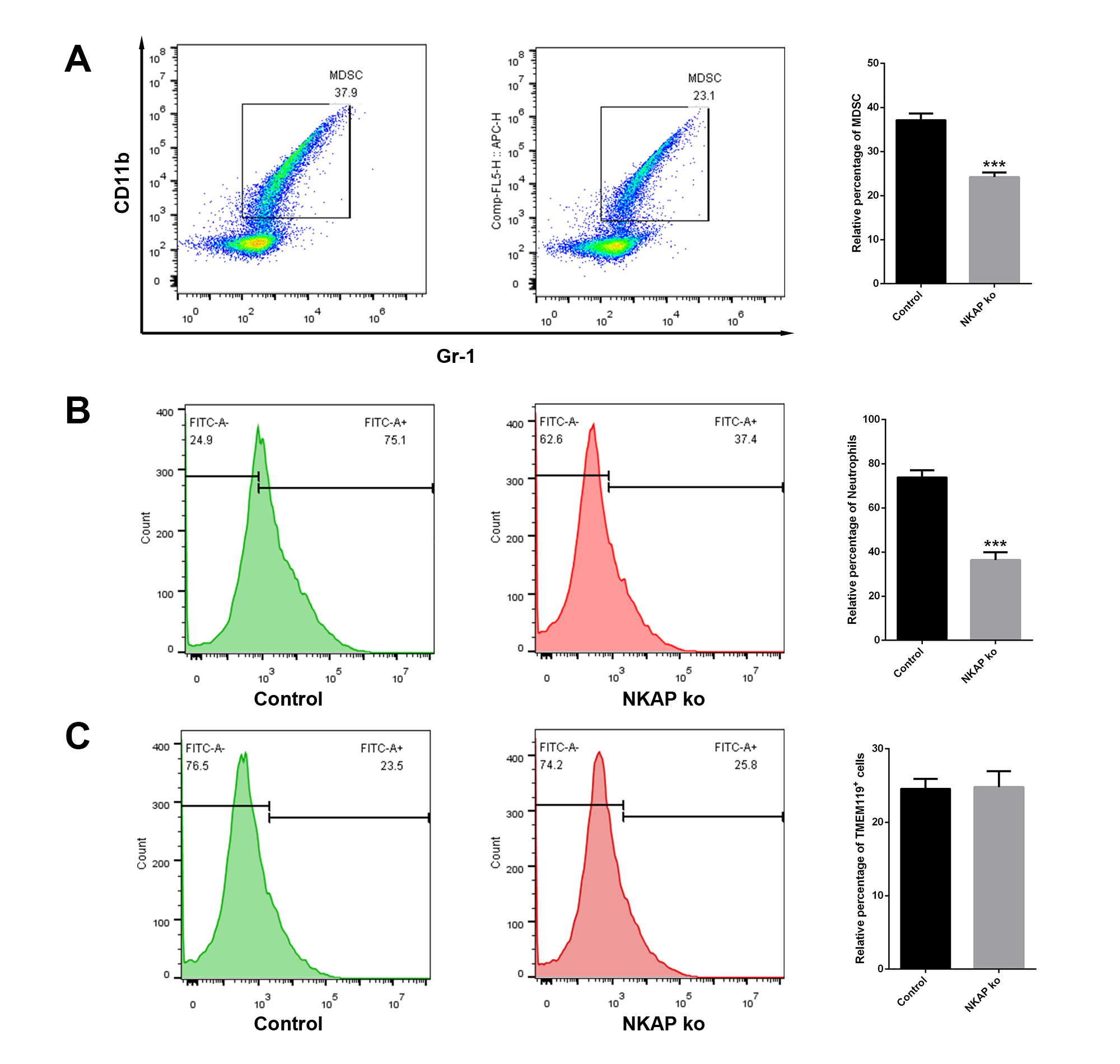

Supplement: Supplementary file 4 — Figure S4. Proportion of myeloid-derived suppressor cells (A) and neutrophils (B) were significantly down-regulated in the NKAP depleted gliomas. C, Percentage of TMEM119 positive microglia was not affected by NKAP knockdown. (TIF 560 kb) [file 13046_2019_1281_MOESM4_ESM.tif]

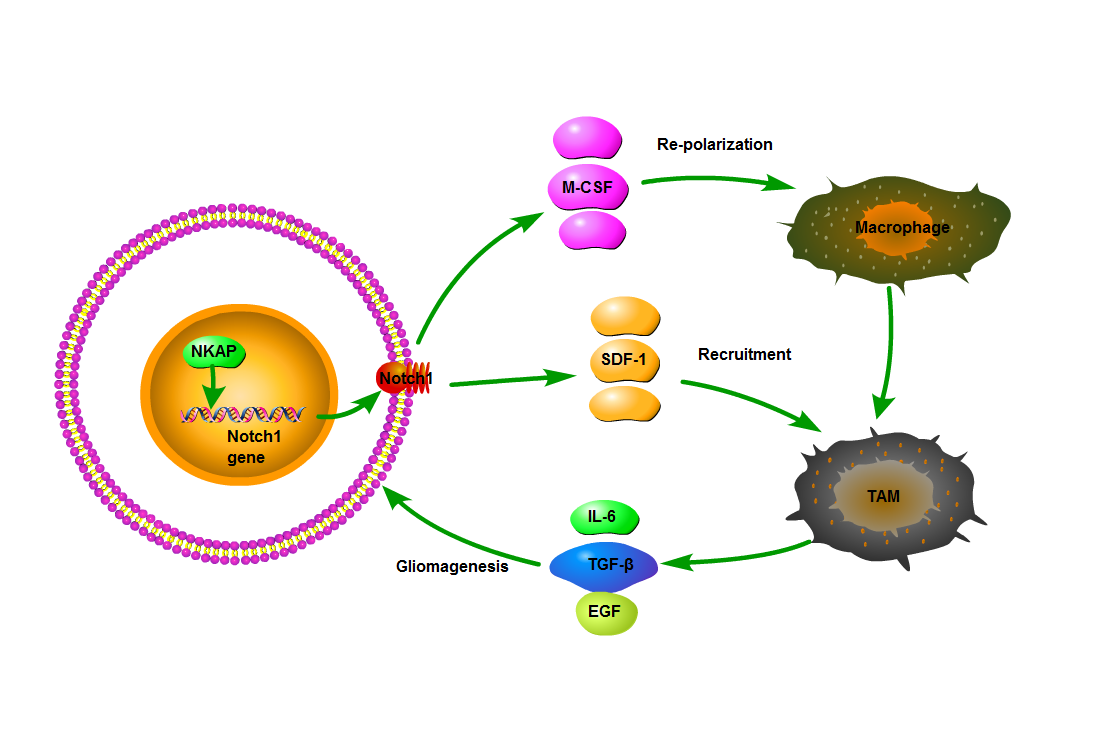

Supplement: Supplementary file 5 — Figure S5. Mechanism map of NKAP in the feedback loop between glioma development and tumor immune microenvironment. (PNG 169 kb) [file 13046_2019_1281_MOESM5_ESM.png]
